# Activity of durvalumab plus olaparib in metastatic castration-resistant prostate cancer in men with and without DNA damage repair mutations

**DOI:** 10.1186/s40425-018-0463-2

**Published:** 2018-12-04

**Authors:** Fatima Karzai, David VanderWeele, Ravi A. Madan, Helen Owens, Lisa M. Cordes, Amy Hankin, Anna Couvillon, Erin Nichols, Marijo Bilusic, Michael L. Beshiri, Kathleen Kelly, Venkatesh Krishnasamy, Sunmin Lee, Min-Jung Lee, Akira Yuno, Jane B. Trepel, Maria J. Merino, Ryan Dittamore, Jennifer Marté, Renee N. Donahue, Jeffrey Schlom, Keith J. Killian, Paul S. Meltzer, Seth M. Steinberg, James L. Gulley, Jung-Min Lee, William L. Dahut

**Affiliations:** 1 Genitourinary Malignancies Branch, Center for Cancer Research, National Cancer Institute, National Institutes of Health, Bethesda, MD USA; 2 Laboratory of Genitourinary Cancer Pathogenesis, Center for Cancer Research, National Cancer Institute, National Institutes of Health, Bethesda, MD USA; 3 Clinical Research Directorate/Clinical Monitoring Research Program, Leidos Biomedical Research, Inc., NCI Campus at Frederick, Frederick, MD USA; 4 Developmental Therapeutics Branch, Center for Cancer Research, National Cancer Institute, National Institutes of Health, Bethesda, MD USA; 5 Genetics Branch, Center for Cancer Research, National Cancer Institute, National Institutes of Health, Bethesda, MD USA; 6 Biostatistics and Data Management Section, National Cancer Institute, National Institutes of Health, Bethesda, MD USA; 7 Women’s Malignancies Branch, Center for Cancer Research, National Cancer Institute, National Institutes of Health, Bethesda, MD USA; 8 Department of Radiology and Imaging Sciences, Center for Cancer Research, National Institutes of Health, Bethesda, MD USA; 9 Laboratory of Pathology, Center for Cancer Research, National Cancer Institute, National Institutes of Health, Bethesda, MD USA; 10 Epic Sciences Inc., San Diego, CA USA; 11 Laboratory of Tumor Immunology and Biology, Center for Cancer Research, National Cancer Institute, National Institutes of Health, Bethesda, MD USA

**Keywords:** Durvalumab, Olaparib, mCRPC, Abiraterone, Enzalutamide, Immunotherapy, Anti-PD-L1, PARP inhibitor

## Abstract

**Background:**

Checkpoint inhibitors have not been effective for prostate cancer as single agents. Durvalumab is a human IgG1-K monoclonal antibody that targets programmed death ligand 1 and is approved by the U.S. Food and Drug Administration for locally advanced or metastatic urothelial cancer and locally advanced, unresectable stage 3 non-small cell lung cancer. Olaparib, a poly (ADP-ribose) polymerase inhibitor, has demonstrated an improvement in median progression-free survival (PFS) in select patients with metastatic castration-resistant prostate cancer (mCRPC). Data from other trials suggest there may be improved activity in men with DNA damage repair (DDR) mutations treated with checkpoint inhibitors. This trial evaluated durvalumab and olaparib in patients with mCRPC with and without somatic or germline DDR mutations.

**Methods:**

Eligible patients had received prior enzalutamide and/or abiraterone. Patients received durvalumab 1500 mg i.v. every 28 days and olaparib 300 mg tablets p.o. every 12 h until disease progression or unacceptable toxicity. All patients had biopsies of metastatic lesions with an evaluation for both germline and somatic mutations.

**Results:**

Seventeen patients received durvalumab and olaparib. Nausea was the only nonhematologic grade 3 or 4 toxicity occurring in > 1 patient (2/17). No patients were taken off trial for toxicity. Median radiographic progression-free survival (rPFS) for all patients is 16.1 months (95% CI: 4.5–16.1 months) with a 12-month rPFS of 51.5% (95% CI: 25.7–72.3%). Activity is seen in patients with alterations in DDR genes, with a median rPFS of 16.1 months (95% CI: 7.8–18.1 months). Nine of 17 (53%) patients had a radiographic and/or PSA response. Patients with fewer peripheral myeloid-derived suppressor cells and with alterations in DDR genes were more likely to respond. Early changes in circulating tumor cell counts and in both innate and adaptive immune characteristics were associated with response.

**Conclusions:**

Durvalumab plus olaparib has acceptable toxicity, and the combination demonstrates efficacy, particularly in men with DDR abnormalities.

**Trial registration:**

ClinicalTrials.gov identifier: NCT02484404.

**Electronic supplementary material:**

The online version of this article (10.1186/s40425-018-0463-2) contains supplementary material, which is available to authorized users.

## Background

Despite success in other solid tumors, checkpoint inhibitors have not shown overall improvements in survival as monotherapy for metastatic castration-resistant prostate cancer (mCRPC). Two phase III trials of ipilimumab failed to meet their endpoint of improved overall survival [1, 2]. Despite this, there were signs of activity, suggesting that a subset of patients may benefit from immunotherapy. Results from patients previously treated with enzalutamide suggest that enzalutamide may increase expression of programmed death ligand 1 (PD-L1) [3]. Indeed, in a separate ongoing trial, 3/12 patients who previously progressed on enzalutamide had objective radiographic responses to programmed cell death protein 1 (PD-1) blockade with pembrolizumab, and 5/28 patients had a prostate-specific antigen (PSA) decline of ≥50% [4]. Mutations in mismatch repair (MMR) genes, one of many pathways involved with DNA damage repair (DDR), are associated with microsatellite instability in advanced prostate cancer and may serve as a possible biomarker of response to immune-blocking antibodies, as seen in other solid tumors. MMR mutations, however, are thought to occur in < 5% of prostate cancer patients [5].

Mutations in other DDR genes appear to occur much more frequently. These include alterations in the homologous recombination repair pathway genes, including *BRCA2* and *ATM*, among others. Alterations in these additional DDR-related genes occur in approximately 20–25% of mCRPC patients, with approximately 12% harboring germline alterations in DDR genes [6, 7]. Alterations in these genes appear to predict response to PARP inhibitors. In a phase II trial of single-agent olaparib, 16/49 patients had either an objective response according to RECIST v.1.1, a reduction of at least 50% in the PSA level or a confirmed reduction in circulating tumor cells (CTCs) (response rate, 33%; 95% CI, 20–48) [8]. The majority of patients who had a response had homologous recombination pathway gene alterations. The phase II KEYNOTE-199 study of single-agent pembrolizumab in docetaxel-refractory mCRPC (NCT02787005) showed patients with somatic mutations in *BRCA1/2* or *ATM* had higher responses rates (9). Whole-exome sequencing was conducted on 6/9 responders with available data, and 4/6 patients had mutations in DDR genes [9].

Mounting evidence from trials in other solid tumors suggests alterations in DDR genes beyond the MMR pathway may also predict response to immunotherapy [10, 11]. There is also increasing rationale for combining PARP inhibition with immunotherapy, even though the mechanism of synergy is not fully understood. Foremost among candidate intracellular pathways is STING (stimulator of interferon genes), an innate immune response activated by cytosolic DNA (perhaps a consequence of DNA damage) that can lead to enhanced interferon (IFN) production [12]. It is evident that, in some patients, mutational burden is associated with response to PD-1/PD-L1 inhibition. It has been suggested that PARP inhibition can potentiate DNA damage and inefficient repair in tumors, and could lead to immunologically relevant mutations [13, 14].

In this study, the PARP inhibitor olaparib is combined with durvalumab to treat mCRPC, regardless of patients’ mutational status. Durvalumab is a human IgG1-K monoclonal antibody that selectively binds human PD-L1 and is approved by the U.S. Food and Drug Administration for the treatment of urothelial cancer and non-small cell lung cancer. In phase I of this study, durvalumab was safely given every 4 weeks at a fixed dose of 1500 mg i.v. in combination with 300 mg of olaparib tablets p.o. every 12 h. This regimen was selected for further study in the phase II cohorts [15]. Here we present findings for the phase 2 mCRPC cohort.

## Results

### Patient characteristics

Between May 2016 and May 2017, 17 patients with mCRPC previously treated with enzalutamide and/or abiraterone were enrolled and treated with durvalumab plus olaparib (Table 1). Five patients had bone-only disease; 12 had bone and soft tissue/visceral disease. Of the 17 patients, 16 (94%) had received enzalutamide and 11 (65%) had received abiraterone. Ten patients (59%) had previously received both enzalutamide and abiraterone. Eleven patients (65%) had prior chemotherapy for metastatic disease. Seven patients (41%) had prior vaccine therapy (2 had prior PROSTVAC, 4 had prior sipuleucel-T, and one had both).

**Table 1:** Baseline characteristics (*n* = 17)

Baseline characteristics	*n* (% or range)
Age	66 (45–79)
Baseline PSA (ng/mL)	79.7 (3.9–2356)
Baseline Hemoglobin (g/dL)	12.2 (9.3–14.5)
Baseline LDH (U/L)	241 (153–351)
Baseline Alkaline Phosphatase (U/L)	80 (56–643)
Baseline CTCs (in 10 mL blood)	12 (0–2107)
Prior treatment with:	
Enzalutamide	16 (94%)
Abiraterone	11 (65%)
Both	10 (59%)
Prior immunotherapy	8 (47%)
Prior chemotherapy	11 (65%)
Gleason score	
< 8	5 (29%)
8–10	12 (71%)
ECOG performance status	
0	2 (12%)
1	14 (82%)
2	1 (6%)
Disease site:	
Bone only	5 (29%)
Bone/soft tissue/viscera	12 (71%)

### Safety

The most common treatment-related grade 3 or 4 adverse events were anemia (4/17; 24%), lymphopenia (2/17; 12%), infection (2/17; 12%), and nausea (2/17; 12%) (Table 2). Four patients had immune-related adverse events (irAEs) of any grade, including 2 with acute onset unilateral hearing loss, one with optic neuritis, and one who developed remitting seronegative symmetrical synovitis with pitting edema (RS3PE). All irAEs were treated with high-dose steroids. Symptoms improved to near complete resolution with high-dose steroids in the patient with optic neuritis and one patient with acute onset unilateral hearing loss, and to complete resolution in the patient with RS3PE. The second patient with acute onset unilateral hearing loss required use of a hearing aid. Durvalumab was discontinued in all patients who developed irAEs, but olaparib was continued. No patients were taken off-study due to toxicity. Patients received a median of 7 cycles of treatment (range: 2–17).

**Table 2:** Adverse events (*n* = 17)

Adverse Event	Grade 1	Grade 2	Grade 3	Grade 4
Hematology				
Anemia	3	2	4	0
Leukopenia	4	1	1	0
Lymphopenia	0	2	1	1
Neutropenia	0	0	1	0
Thrombocytopenia	3	0	1	0
Gastrointestinal Disorders				
Abdominal pain	1	0	0	0
Anorexia	1	1	0	0
AST increased	1	0	0	0
Bloating	0	1	0	0
Dysgeusia	0	1	0	0
Dyspepsia	2	0	0	0
Diarrhea	6	3	0	0
Nausea	8	1	2	0
Oral mucositis	0	1	1	0
Vomiting	6	0	1	0
Cardiovascular				
Hypertension	0	0	1	0
Infection				
Lung	0	0	1	0
Tenosynovitis	0	0	1	0
Urinary tract	0	1	0	0
Endocrine and Chemistry				
Hypothyroidism	0	1	0	0
Nervous System				
Dizziness	1	0	0	0
Headache	1	0	0	0
Paresthesia	1	0	0	0
Syncope	0	0	1	0
Respiratory, Thoracic, and Mediastinal				
Dyspnea	1	0	0	0
Cough	1	1	0	0
Eye Disorders				
Blurred vision	1	0	0	0
Optic nerve disorder	0	1	0	0
Ear and Labyrinth Disorders				
Hearing impairment	0	1	1	0
Tinnitus	1	0	0	0
General				
Fatigue	1	2	1	0
Edema, limbs	1	0	0	0
Localized edema	1	0	0	0
Pain, extremity	0	1	0	0
Weight loss	1	0	0	0
Musculoskeletal and Connective Tissue				
Arthralgia	2	0	0	0
Arthritis	0	1	0	0
Myalgia	2	0	0	0
Musculoskeletal/connective tissue, leg cramps	1	1	0	0
Musculoskeletal/connective tissue, muscle cramps	0	0	1	0
Muscle weakness, lower limb	0	0	1	0
Skin, Subcutaneous Tissue				
Erythema	1	0	0	0
Pruritis	1	0	0	0
Rash, maculopapular	2	0	0	0
Metabolism and Nutrition				
Dehydration	0	1	0	0

### Treatment outcomes and immune predictors of response

**Fig. 1:**
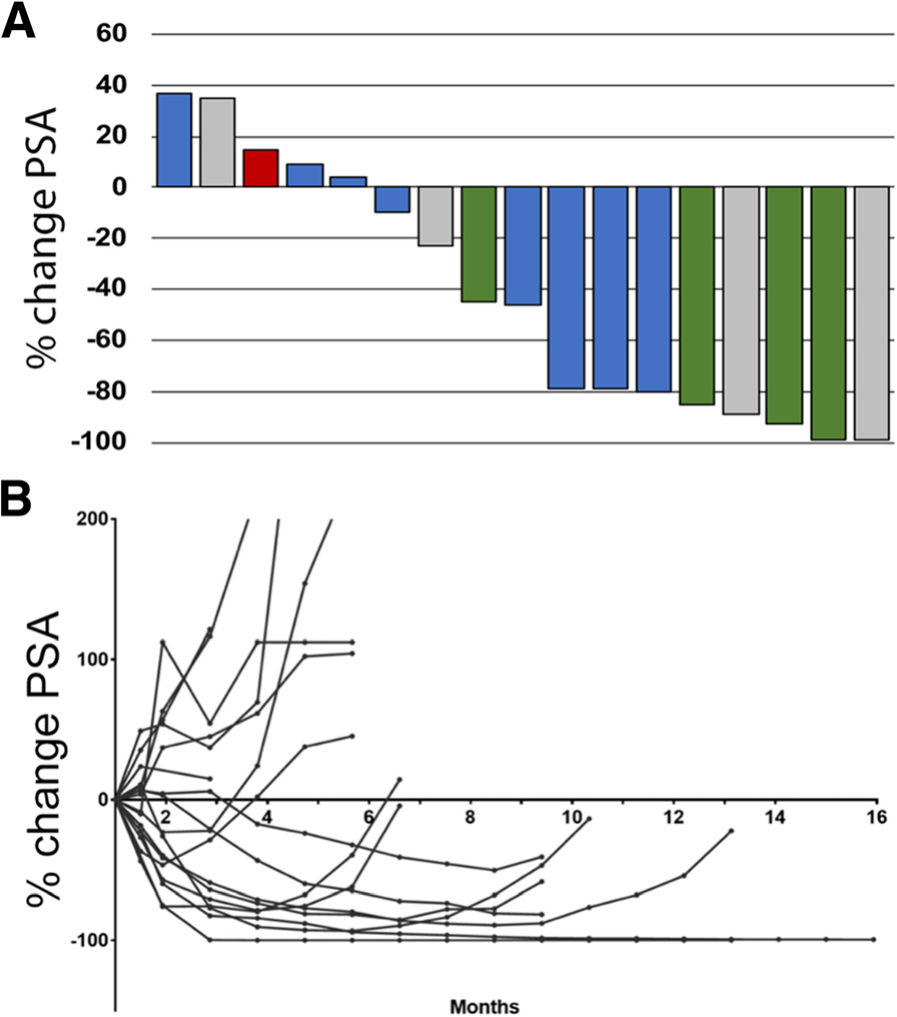
PSA Response. **a** Waterfall plot demonstrating maximum decline in PSA for each patient. Bar colors represent radiographic response by RECIST criteria: green, partial response; blue, stable disease; red, progressive disease; gray, not assessable (bone-only disease). **b** Spider plot of PSA responses over time

**Fig. 2:**
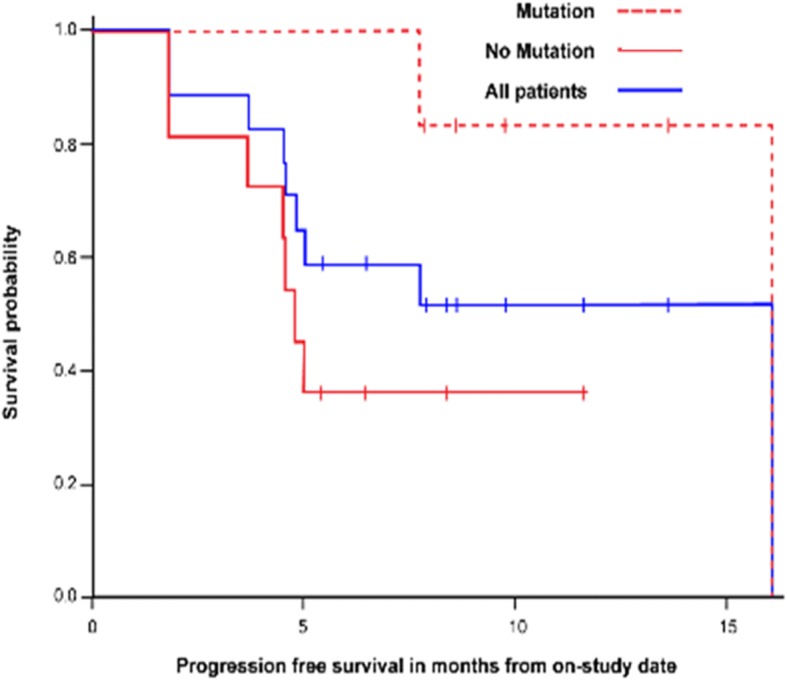
Progression-Free Survival. At median potential follow-up of 9.7 months, radiographic PFS for all patients with durvalumab plus olaparib (*n* = 17) is 16.1 months

Nine of 17 patients (53%) had a PSA decline of ≥50% (defined as responders). Of those 9 patients, 4 had a radiographic response per RECIST v.1.1 (Fig. 1a, b). For all patients, the 12-month PFS is 51.5% (95% CI: 25.7–72.3%). The median radiographic progression-free survival (rPFS) of patients with alterations in DDR genes was 16.1 months (95% CI: 7.8–18.1 months) (Fig. 2), with 12-month PFS probability of 83.3% (95% CI: 27.3–94.5%) compared with a 12-month probability of 36.4% (95% CI: 11.2–62.7%) for those without mutations; exact *p* = 0.031.

### Early indicators of response

**Fig. 3:**
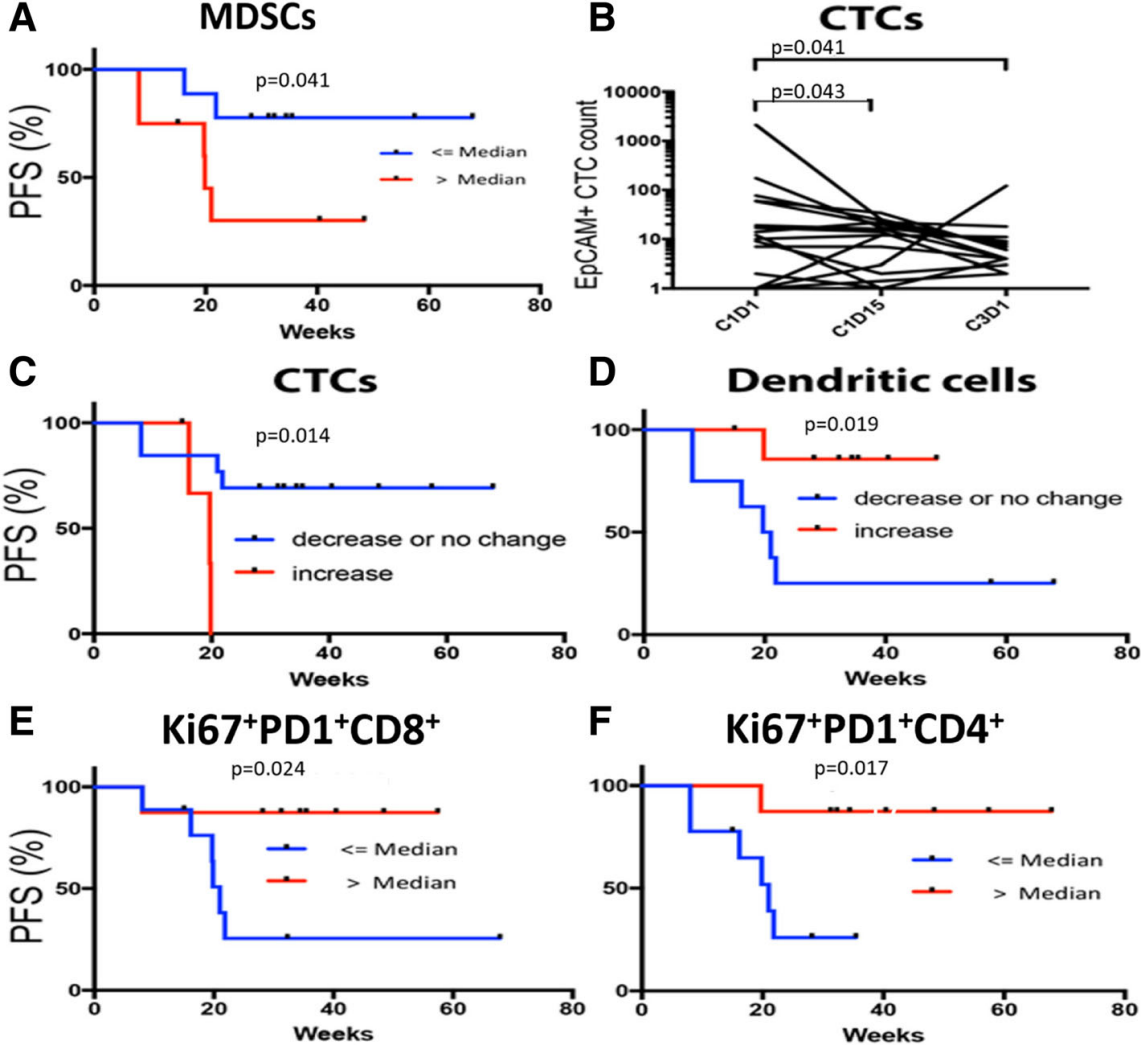
Early Markers of Response Are Associated with Progression-Free Survival. **a** Kaplan-Meier curve showing that patients with <= median percentage of MDSCs at baseline had prolonged PFS. **b** Change in CTC numeration from C1D1 to C3D1. **c** Decrease or no change in in CTC count from C1D1 to C1D15 correlated with increased PFS. **d** Kaplan-Meier curve demonstrating that increased DC maturity (as demonstrated by CD83 expression on CD141^+^ mDCs from baseline to C1D15) was associated with prolonged PFS. **e** Kaplan-Meier curve demonstrating that patients with > median percentage of K67^+^PD^−^1^+^CD8^+^ T cells among total CD8^+^ T cells at C1D15 had prolonged PFS. **f** Kaplan-Meier curve demonstrating that patients with > median percentage of K67^+^PD^−^ 1^+^CD4^+^ T cells among total CD4^+^ T cells at C3D1 had prolonged PFS

Patients’ baseline fraction of myeloid-derived suppressor cells (MDSCs) correlated with response to therapy. Patients whose percentage of MDSCs among total viable cells at baseline was ≤ the median had prolonged PFS (*p* = 0.041) (Fig. 3a). As with multiple chemotherapy trials, circulating tumor cell (CTC) response was an early predictor of benefit (Fig. 3b, c) for this immunotherapy trial. EpCAM^+^ CTCs were assessed at cycle 1 day 1 (C1D1), C1D15, and C3D1 in all 17 patients. Baseline CTCs varied among the 17 patients (0–2107 cells/10 mL of blood). The CTC count decreased or was unchanged in response to therapy in 13/17 patients (76%) at C1D15 and in 12/17 patients (71%) at C3D1 (Fig. 3b, Table 3). Patients with no change or a decrease in CTCs from C1D1 to C1D15 in response to treatment had prolonged PFS compared with those in whom CTCs increased (Fig. 3c).

**Table 3:** CTC enumeration; The number of CTCs in 10-ml blood

Patient	C1D1	C1D15	C3D1
#1	59	20	8
#2	7	7	4
#3	77	24	4
#4	9	2	3
#5	2	1	1
#6	1	0	2
#7	2107	25	7
#8	10	12	0
#9	12	1	4
#10	19	16	4
#11	60	34	6
#12	1	12	11
#13	0	0	3
#14	175	17	9
#15	14	22	18
#16	17	14	2
#17	1	3	122

Early immune responses also were associated with benefit. Dendritic cells (DCs) that express CD141 produce IFN-γ and support CD4^+^ T-cell polarization to a Th1 phenotype [16]. Expression of CD83 on CD141^+^ DCs is a functional marker of fully mature DCs (mDCs) that present antigen and induce T cell-mediated immune responses [17]. In our cohort, patients with increased expression of CD83 on CD141^+^ mDCs from C1D1 to C1D15 had prolonged PFS (Fig. 3d).

Changes in CD8^+^ and CD4^+^ cell populations also predicted response. Patients with > median percentage of Ki67^+^PD-1^+^ cells among total CD8^+^ T cells in response to therapy had prolonged PFS (Fig. 3e), as did patients with > median percentage of Ki67^+^PD-1^+^ cells among total CD4^+^ T cells (Fig. 3f). Analysis of expression of HLA-DR, another T-cell activation marker, showed that patients with > median percentage of Ki67^+^HLA-DR CD8^+^ and CD4^+^ T cells at C3D1 had prolonged PFS.

### Molecular characteristics of responders

**Fig. 4:**
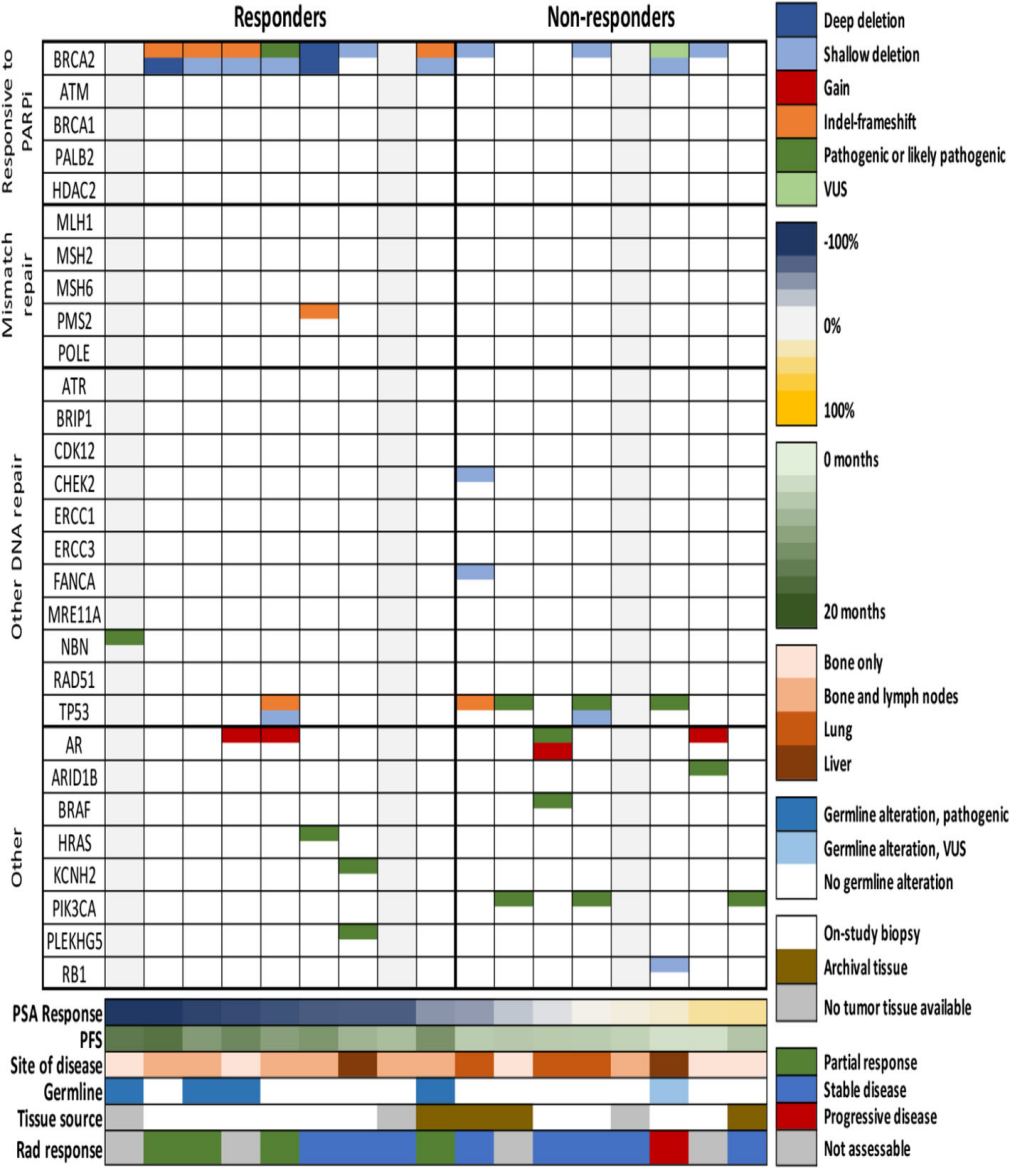
Genomic Alterations. Presence or absence of alterations in DDR and other significant genes. Genomic data are from OncoVar sequencing, a capture-based sequencing panel of 500 cancer-associated genes. Copy number calls are based on read depth and minor allele frequency in the OncoVar sequencing results. All patients had germline sequencing performed. As indicated, 3 patients had insufficient tumor tissue on biopsy and no archival tissue available

Using a sequencing panel targeting 500 cancer-associated genes, we performed genomic analysis of germline DNA for all patients and tumor DNA for 14/17 patients (Fig. 4). Four responders harbored germline alterations in DDR genes: one with a known deleterious mutation in *NBN* and 3 with frameshift indels in *BRCA2*. The patients with germline *BRCA2* indels had tumor tissue available which demonstrated somatic deletion of the second allele.

Two additional responders had homozygous somatic alterations in *BRCA2*: deletion of one allele and the second allele affected by deleterious nonsynonymous mutation or deletion. One patient also harbored a frameshift indel of *PMS2*, an MMR gene, though the second allele appeared to be intact and there was no evidence of a hypermutation phenotype. Twoother responders had no detected DDR gene biomarker of response. Of these twoone had shallow loss of *BRCA2* with no alteration detected in the other allele, and one had no tumor tissue available for analysis. Three patients have ongoing responses of > 12 months: one with a germline *NBN* mutation, one with somatic deletion of both copies of *BRCA2*, and one with germline indel in BRCA2 and somatic loss of the remaining allele.

PD-L1 expression by immunohistochemical (IHC) staining was evaluated in 5 patients who had tumor tissue available. Of these, 1 patient was positive for PD-L1 expression (3–5%). This patient was defined as a responder (PSA decline of ≥50%). Of the remaining 4 patients, 3 were negative for PD-L1 expression by IHC and all 3 were responders. The remaining patient was weakly positive (1–2%) but was a non-responder. In another CTC analysis by Epic Sciences (Epic Sciences Inc., San Diego, CA, USA) baseline CTCs were evaluated for PD-L1 expression in 10 patients. Two of these 10 patients also had PD-L1 expression evaluated by IHC. No patient was found to have PD-L1 positivity.

Lastly, the feasibility of measuring PD-L1 by flow cytometry in peripheral immune subsets following treatment with durvalumab was determined. In vitro binding studies using healthy donor peripheral blood mononuclear cells (PBMCs) showed that anti-PD-L1 clone MIH1 does not complete with durvalumab and can be used to detect PD-L1 after durvalumab exposure (Additional file 1: Table S3). PD-L1 expression in available PBMCs was then evaluated in 4 patients after treatment with durvalumab and olaparib; similar frequencies of PD-L1 were detected as has previously been described for patients with advanced cancer [18]. (Table 4-patient 3 was a non-responder and patients 7, 11, and 14 were responders).

**Table 4:** Percentage of 9 Classic Subsets Expressing PD-L1

PT	PT3	PT7	PT11		PT14	Median of PTs	HD1	HD2	Median of HDs
Days Post Tx	D98	D561	D456	D596	D448				
PD-L1 + CD4	1.9	1.2	1.5	1.6	1.5	1.5	0.7	0.5	0.6
PD-L1 + CD8	0.9	0.9	0.6	1.1	1.8	0.9	1.6	0.8	1.2
PD-L1 + Treg	0.3	0.1	0.8	0.6	0.6	0.6	0.2	0.1	0.2
PD-L1 + NK	3.1	1.8	1.3	1.3	1.6	1.6	1.0	0.5	0.8
PD-L1 + NKT	38.2	1.5	0.8	0.8	1.6	1.5	2.2	0.9	1.5
PD-L1 + B cells	19.45	6.75	14.12	12.83	13.14	13.1	29.32	14.00	21.7
PD-L1 + cDc	25.6	2.2	2.5	3.3	5.5	3.3	4.3	1.3	2.8
PD-L1 + pDc	< 0.01	< 0.01	< 0.01	< 0.01	< 0.01	< 0.01	< 0.01	< 0.01	< 0.01
PD-L1 + MDSC	19.34	11.81	10.12	11.42	15.11	11.8	4.37	5.76	5.1

Expression of PD-L1 in 9 classic subsets was measured by flow cytometry in 4 patients, where PBMCs were available, after treatment with durvalumab and olaparib. Results are displayed as % of classic subsets that express PD-L1. *cDC* conventional dendritic cells, *MDSC* myeloid derived suppressor cell, *NK* natural killer, *pDC* plasmacytoid DC, *PD-L1* programmed cell death ligand-1, *Tregs* regulatory T cells

## Discussion

The preliminary PFS data and response rate for this cohort of mCRPC patients treated with durvalumab plus olaparib indicate deep and sustained responses with this therapy combination, with 53% of patients having radiographic and/or PSA responses, median PSA decline of 85% among the 9 responders, and median duration of response of 16.1 months in those patients with mutations in DDR genes. One third of responders harbored germline mutations in DDR genes, one third had detectable biallelic somatic alterations in DDR genes, and one third had neither.

Mutational burden, DDR status, prior therapies, and other variables can critically affect the cancer-immune set point, the peripheral immune phenotype, and response to therapy [19]. The data presented here suggest that durvalumab plus olaparib can affect both innate and adaptive immunity in patients with mCRPC, and that engagement of these 2 types of immunity may be associated with prolonged PFS. Our data also suggest that durvalumab plus olaparib for mCRPC may promote dendritic cell (DC) maturation, enhance CD4^+^ T-cell activity, and reactivate CD8^+^ T-cell antitumor immunity.

Early immune responses were associated with benefit. It has been suggested that DCs must be optimally stimulated to overcome the immunosuppressive tumor microenvironment and achieve robust and durable responses to immunotherapy [16]. Durvalumab plus olaparib may enhance maturation of DCs into CD83^+^CD141^+^ mDCs that are competent to present antigen and direct adaptive immune responses. Therefore, our finding of increased mDCs at C1D15 is consistent with increased activation of CD4^+^ and CD8^+^ T cells in response to durvalumab plus olaparib. Dendritic cells (DCs) that express CD141 produce IFN-γ and support CD4^+^ T-cell polarization to a Th1 phenotype [16]. Expression of the functional marker CD83 on CD141^+^ DCs is associated with fully mDCs that present antigen and induce T cell-mediated immune responses [17]. In our cohort, patients with increased expression of CD83 on CD141^+^ mDCs from C1D1 to C1D15 had prolonged PFS (Fig. 3d).

Changes in CD8^+^ and CD4^+^ cell populations also predicted response. Patients with > median percentage of Ki67^+^PD-1^+^ cells among total CD8^+^ T cells in response to therapy had prolonged PFS (Fig. 3e), as did patients with > median percentage of Ki67^+^PD-1^+^ cells among total CD4^+^ T cells (Fig. 3f). Analysis of expression of HLA-DR, another T-cell activation marker, showed that patients with > median percentage of Ki67^+^HLA-DR CD8^+^ and CD4^+^ T cells at C3D1 had prolonged PFS.

In two recent studies, patients with melanoma [20] or non-small cell lung cancer [21] had increased levels of Ki67^+^PD-1^+^CD8^+^ T cells in peripheral blood after treatment with a PD-1-targeting agent, which was considered a potential indicator of activated tumor-specific CD8^+^ T cells. Kamphorst et al. [21] propose that early PD-1^+^CD8^+^ T-cell responses may be associated with clinical outcome. They note that it may be difficult to detect cycling PD-1^+^CD8^+^ T cells in peripheral blood due to their transient appearance and the timing of peripheral blood analyses. In our study, our time points appear to facilitate detection of this transient immune population. Furthermore, at C1D15 we see apparent associations of Ki67^+^PD-1^+^CD8^+^ T cells with improved PFS. At C3D1 the association of Ki67^+^CD8^+^ T cells with PFS is no longer apparent, but 2 new associations appear to correspond with PFS: Ki67^+^HLA-DR^+^CD8^+^ T cells and Ki67^+^PD-1^+^CD4^+^ T cells.

The efficacy seen in this trial may be due to synergy between the 2 agents. Recent data show that DNA damage plays a role in priming the type I IFN system, where DNA damage results in enhanced production of type I IFNs via the cytosolic DNA sensor STING, which can prime the innate immune system for an amplified response [22]. STING is involved in controlling the transcription of host defense genes, including type I IFNs, following recognition of DNA in the cell cytosol [12]. Leaked DNA in the cytosol binds to cyclic GMP-AMP synthase (cGAS), leading to an upregulation of the STING pathway. This in turn activates type I IFNs and cytosolic DNA sensors such as cGAS [23], which potently prime antitumor T cells [12].

PARP inhibition may also render tumor cells more amenable to immune attack through immunogenic modulation, as has been demonstrated with other targeted therapies [24]. Preclinical in vivo and in vitro breast cancer models have shown that PARP inhibition can upregulate PD-L1 by inactivating GSK3β, and that subsequent blockade of PD-L1 resensitizes PARP inhibitor-treated cells to T-cell killing [25]. Additionally, recent data suggest that expression of PD-L1 is upregulated in response to DNA double-strand breaks, and that in *BRCA*2-depleted cells, PARP inhibition upregulates PD-L1 expression [26]. Combination therapy may be effective, regardless of DDR mutational status, if DNA damage signaling in tumors is down-regulated, thus increasing PD-L1 expression in tumors.

In this study, 2/3 of responders had DDR gene alterations that would predict response to olaparib. Therefore, the depth and duration of response in this trial may simply be an enhancement and prolongation of olaparib’s efficacy when given in combination with durvalumab. However, DDR alteration biomarkers were identified in only 2/3 of responders compared to 88% of patients in the TOPARP-A trial [8]. Emerging data in metastatic urothelial cancer suggest that tumor mutational burden is associated with DNA replication and DDR pathways [11]. Tumors with mutations in DDR genes had higher tumor mutational burden and response rates to PD-L1 blockade with atezolizumab [11]. The deep and sustained responses seen with the combination of durvalumab plus olaparib may reflect the possible association between DDR mutational status and increased potential for immunogenicity [11].

Some of the DDR biomarker-negative responders had shallow deletions of *BRCA2*. Given the responses, one hypothesis is that the shallow deletion in these responders reflects deep deletion in a subset of tumor cells rather than shallow deletion in all cells, as has been previously proposed [27]. The checkpoint inhibitor may extend this response to additional, unaltered tumor cells. In the TOPARP-A trial, the only responder with a shallow *BRCA2* deletion also had a deletion in *PALB2*.

The range of immune-related toxicities seen in this study raises the possibility of additive or synergistic toxicity. In addition to myelosuppression and nausea attributable to olaparib, 4/17 patients (24%) experienced side effects consistent with irAEs, including sudden-onset hearing loss, optic neuritis, and a case of RS3PE of the hands and upper extremities, all successfully treated with transient steroids. While these toxicities have been reported with PD-L1 inhibition, the high number of apparent irAEs in such a small cohort is noteworthy given the broader clinical experience with these agents [28–30]. It is unclear if these toxicities are the byproduct of antitumor immunologic synergy or if PARP inhibitors immunologically modify off-target healthy tissue, perhaps rendering them more vulnerable to immunologic attack.

Though not detected in this cohort, DNA MMR mutations resulting in microsatellite instability are also associated with higher mutational burden and neoantigens [31]. The only MMR alteration we noted was an indel in *PMS2* in a patient harboring *BRCA2* homozygous deletion. We did not see any evidence of a hypermutation phenotype in this or any other patient. Using genomic analysis of mCRPC samples, recent data has identified a subtype of mutant tumors in advanced prostate cancer with biallelic loss of *CDK12* [32]. *CDK12* biallelic loss is associated with increased immunogenicity due to higher neoantigen burden compared to other molecular subtypes of prostate cancer and is mutually exclusive with mutations in DDR [32]. Patients with *CDK12* mutations may benefit from immune therapies. *CDK12* mutations were not detect in this cohort of patients.

The limitations of our study, such as a small cohort of patients, require cautious interpretation of findings. This is a single-arm study in which all patients receive the same combination therapy. It is not clear to what extent there is synergy of the combination vs. additive effects of 2 individual drugs. In this small patient cohort, pharmacodynamic markers are exploratory and hypothesis-generating. Thus, we found that baseline immune characteristics, in addition to alterations of DDR genes, may predict outcomes in patients treated with durvalumab plus olaparib. In addition, early changes in immune characteristics may be early indicators of clinical outcome. Preliminary data are being reported due to the nature of results seen thus far, particularly the sustained PSA responses. To further investigate combination therapy with durvalumab plus olaparib, we plan to expand the mCRPC cohort up to an additional 65 patients to obtain more detailed response information and conduct additional correlative studies. Patients will continue to undergo mandatory on-study biopsies of a metastatic site of disease, with additional biopsies planned. The expansion cohort will enable in-depth correlative analysis of this combination therapy to better define a potential therapeutic synergy and facilitate biomarker development.

## Conclusions

The clinical development of inhibitors of PARP and PD-1/PD-L1 has had a substantial impact on the treatment of advanced malignancies. This is the first study to demonstrate activity for the combination of these agents in prostate cancer patients without biallelic inactivation in DDR pathways, and deep responses in patients with known mutations. While the study is limited by a small patient cohort, the 12-month PFS is 51.5% in patients with advanced metastatic disease, > 50% of whom are taxane-refractory. The future of treatment for mCRPC may take us beyond androgen suppression to combination therapies such as PARP inhibition plus immunotherapy.

## Patients and methods

### Patient selection

This phase II, open-label trial includes multiple cohorts. Results of the phase I dose-escalation study in women’s cancers were reported separately [15]. In the phase II mCRPC cohort, eligible patients had histopathologically confirmed mCRPC that had progressed after previous treatment with enzalutamide and/or abiraterone and had at least one lesion (soft tissue/viscera or bone) deemed safe to biopsy. Patients were 18 years or older and had an Eastern Cooperative Oncology Group performance status of 0–2 and adequate organ function. There were no limitations on previous standard therapies, including previous chemotherapy for mCRPC or metastatic castration-sensitive prostate cancer; however, previous use of immune checkpoint monoclonal antibodies or PARP inhibitors was excluded. Patient selection was not based on mutational status (somatic and/or germline) or other biomarkers. Use of steroids (prednisone or equivalent corticosteroid) exceeding 10 mg/day was not allowed. All patients gave written informed consent in accordance with federal, state, and institutional guidelines. This study was approved by the Institutional Review Board of the Center for Cancer Research, National Cancer Institute, and is registered with ClinicalTrials.gov (NCT02484404).

### Study design and correlatives

This is a single-institution, single-arm, open-label clinical trial to determine the clinical efficacy of durvalumab plus olaparib in an unselected population with advanced prostate cancer. Secondary endpoints include overall response rate by RECIST v.1.1, safety, duration of response, and PSA response. One cycle was defined as 28 days. Tissue obtained from mandatory on-study biopsies was analyzed by the OncoVar assay, a 500-cancer gene hybrid capture sequencing panel. The gene panel coding region is sequenced to an average coverage of >100x. DNA from saliva served as a germline reference. The DNA was used to construct a genomic DNA library, in-solution hybridization to RNA baits targeting the genes, and single-molecule sequencing of the partitioned DNA library. Mutational analysis was performed using Mutect 2 [33] with visual confirmation using IGV [34]. Cnvkit2 [35] was used to make copy number estimates based on read depth and minor allele frequencies in the OncoVar results, using default log2 ratio thresholds of <− 1.1 and < − 0.25 for deep and shallow deletions, respectively. Nexus Copy Number (BioDiscovery, El Segundo, CA) was used for visual confirmation of copy number estimatesPeripheral blood mononuclear cells were assessed using multiparametric flow cytometry for immune subsets. RNA extracted from peripheral blood was analyzed for expression of immune genes and control genes.

### Treatment plan and toxicity evaluation

Patients were given a one-hour i.v. infusion of durvalumab 1500 mg every 28 days, plus olaparib tablets 300 mg every 12 h on a 28-day cycle. Treatment continued until confirmed disease progression, unacceptable toxicity, or patient withdrawal from study. Treatment was delayed or discontinued following specified adverse events of different grades, according to protocol guidelines. Dose reductions and dose interruptions were allowed per protocol. No intra-dose escalation was permitted. Safety was assessed at each monthly visit. Adverse events were classified and graded per the NCI Common Terminology Criteria for Adverse Events v.4.0. The study drugs did not require any premedications. Radiographic tumor assessments with bone scan and CT scan of the chest, abdomen, and pelvis were required at baseline, at 8 weeks, and then every 12 weeks. Response and progression were evaluated according to RECIST v.1.1 [36]. Radiographic progression was defined as (a) the first occurrence of 2 new lesions on bone scan, or (b) progression of measurable disease by RECIST v.1.1 as per the Prostate Cancer Clinical Trials Working Group 2 [37]. Disease progression was determined by clinical and radiographic criteria without evaluating PSA [37].

### Statistical methods

Patients were enrolled in a pilot/phase II evaluation using the recommended phase II dose to determine if the drug combination was associated with improved PFS (70% PFS vs. an estimated 50% PFS at 4 months). The study was designed to enroll 25 patients in order to have 80% power to determine an improvement in 4-month PFS, with a one-sided 0.10 alpha level test, using the Brookmeyer and Crowley method in a preliminary cohort of patients [38]. In addition, an early stopping rule was implemented: if, after 12 patients were enrolled and followed for 6 months, the 4-month PFS was ≤50%, the mCRPC cohort would enroll no more patients. PFS was evaluated for all patients by the Kaplan-Meier method, starting from the on-study date until date of progression or death without progression, with censoring for removal from study because of patient preference or investigator discretion. The significance of the difference between Kaplan-Meier curves based on mutational status was determined by an exact 2-tailed log-rank test.

### Pharmacodynamic studies

CTCs enumerated in Fig. 3b and c were identified as viable, nucleated CD45^−^EpCAM^+^ cells, as previously described [39–41]. For immune subset analysis, blood samples were collected, processed, and analyzed as previously described [40–43]. MDSCs were defined as CD3^−^CD19^−^CD56^−^HLA-DR^−^CD11b^+^CD33^+^ cells, as previously described [44]. Details of the reagents used are provided in Additional file 2. All analyses were performed using multiparametric flow cytometry (MACSQuant; Miltenyi Biotec, Bergisch Gladbach, Germany), and data were analyzed using FlowJo software ​v.10.0.7 (FlowJo, LLC, Ashland, OR).

### Immunohistochemistry

IHC staining was preceded by antigen retrieval (20 min), achieved by steaming deparaffinized and rehydrated sections in Tris-EDTA, pH 9. Sections were incubated with a primary antibody rabbit anti PD-L1 clone E1L3N (Cell Signaling. Danvers, MA) 1:50 dilution overnight at + 4 degrees Celsius; Antibody binding was detected by an envision using peroxidase and DAB, diaminobenzidine. The positive control used for PD-L1 IHC was human mature placenta, and MCF-7 with negative PD-L1 protein expression.

For PD-1, a primary anti mouse PD-1 clone EH33 (Cell Signaling.Danvers, MA) 1: 100 dilutions was utilized with the procedure described above, and using lymph node as a positive control.

The immunohistochemical results were PD-L1 considered positive when membranous tumor cell staining was observed in at least 1% of the tumor cells according to the Keynote 010 study [45].

### Flow cytometry

PD-L1 expression was evaluated in peripheral immune subsets (CD4+ T cells, CD8+ T cells, Regulatory T cells, natural killer (NK) cells, NK-T cells, B cells, conventional dendritic cells, plasmacytoid dendritic cells, and myeloid derived suppressor cells) by flow cytometry using the clone MIH1 [18, 46]. PBMC isolated from 2 healthy donors (HD) were thawed, and then pre-incubated for 40 min with the indicated concentrations of durvalumab or IgG1 control prior to multiparametric stains of PD-L1 to detect PD-L1 within the various immune cell types. Cells that were not pre-incubated with the isotype control or durvalumab also served as controls. PD-L1 was detectable and measured at a similar frequency within immune cell subsets (data shown as percentage of CD4, CD8, cDC and B cells expressing PD-L1), regardless of whether the PBMC were preincubated with the IgG1 isotype control or durvalumab.

## Additional file

Additional file 1: **Figure S1.** Gating strategy. **A** Gating strategy for MDSCs; After gating on the single viable cell population MDSCs were identified as the CD3-CD19-CD56-HLA-DR-CD11b + CD33+ cell population. **B** Gating strategy for circulating tumor cells (CTCs) after EpCAM enrichment CTCs were identified as nucleated, viable CD45-EpCAM+ cells. **C** Gating strategy for CD1c + mDC1 subset and CD83 expression; viable CD3-CD19-CD56-CD11c + HLA-DR + CD1c + cells were further identified as CD1c + mDC1 and CD83 expression was measured. **D** Gating strategy for Ki67 + PD-1 + CD8+ T cells and Ki67 + PD-1 + CD4+ T cells. **Table S2.** Median and IQR of the immunological correlates. **Table S3.** Percentage of Classic Subsets Expressing PD-L1. PD-L1 clone MIH-1 used to detect surface expression of PD-L1 in immune cell subsets does not compete for binding with durvalumab. These results demonstrate that the PD-L1 clone (MIH-1) does not compete for binding with durvalumab in PBMC and can thus be used to measure PD-L1 expression in patients treated with durvalumab. (ZIP 3994 kb)

Additional file 2: A. Immunophenotyping of myeloid-derived suppressor cells (MDSCs). B. Statistical analysis of pharmacodynamic endpoints. (DOCX 18 kb)

## Abbreviations

C: Cycle
cDC: Conventional dendritic cells
cGAS: Cyclic GMP-AMP synthase
CTC: Circulating tumor cell
D: Day
DC: Dendritic cell
DDR: DNA damage repair
IFN: Interferon
irAE: Immune-related adverse event
mCRPC: Metastatic castration-resistant prostate cancer
mDC: Mature dendritic cell
MDSC: Myeloid derived suppressor cell
MDSC: Myeloid-derived suppressor cell
MMR: Mismatch repair
NK: Natural killer
PD-1: Programmed cell death protein 1
pDC: Plasmacytoid DC
PD-L1: Programmed death-ligand 1
PSA: Prostate-specific antigen
rPFS: Radiographic progression-free survival
RS3PE: Remitting seronegative symmetrical synovitis with pitting edema
STING: Stimulator of interferon genes
Tregs: Regulatory T cells
