# Supplementary material for: Activity of durvalumab plus olaparib in metastatic castration-resistant prostate cancer in men with and without DNA damage repair mutations
Source: J Immunother Cancer. 2018 Dec 4;6:141. doi: 10.1186/s40425-018-0463-2 (PMC6280368; doi:10.1186/s40425-018-0463-2)
Supplement: Supplementary file 1 — Figure S1. Gating strategy. A Gating strategy for MDSCs; After gating on the single viable cell population MDSCs were identified as the CD3-CD19-CD56-HLA-DR-CD11b + CD33+ cell population. B Gating strategy for circulating tumor cells (CTCs) after EpCAM enrichment CTCs were identified as nucleated, viable CD45-EpCAM+ cells. C Gating strategy for CD1c + mDC1 subset and CD83 expression; viable CD3-CD19-CD56-CD11c + HLA-DR + CD1c + cells were further identified as CD1c + mDC1 and CD83 expression was measured. D Gating strategy for Ki67 + PD-1 + CD8+ T cells and Ki67 + PD-1 + CD4+ T cells. Table S2. Median and IQR of the immunological correlates. Table S3. Percentage of Classic Subsets Expressing PD-L1. PD-L1 clone MIH-1 used to detect surface expression of PD-L1 in immune cell subsets does not compete for binding with durvalumab. These results demonstrate that the PD-L1 clone (MIH-1) does not compete for binding with durvalumab in PBMC and can thus be used to measure PD-L1 expression in patients treated with durvalumab. (ZIP 3994 kb) [file 40425_2018_463_MOESM1_ESM.zip › Table S2 Median and IQR of the immunological correlates.docx]

**Table S2** Median and IQR of the immunological correlates

|  | 3A | 3E | 3F |
| --- | --- | --- | --- |
| Biomarker | % MDSCs / viable PBMCs | % Ki67+PD-1+CD8+ T cells / CD8+ T cells | % Ki67+PD-1+CD4+ T cells / CD4+ T  cells |
| Time point | C1D1 | C1D15 | C3D1 |
| Median % | 1.26 | 1.73 | 2.00 |
| IQR % | 0.53-1.84 | 0.96-2.68 | 1.72-2.81 |

**Table S3** Percentage of Classic Subsets Expressing PD-L1

**
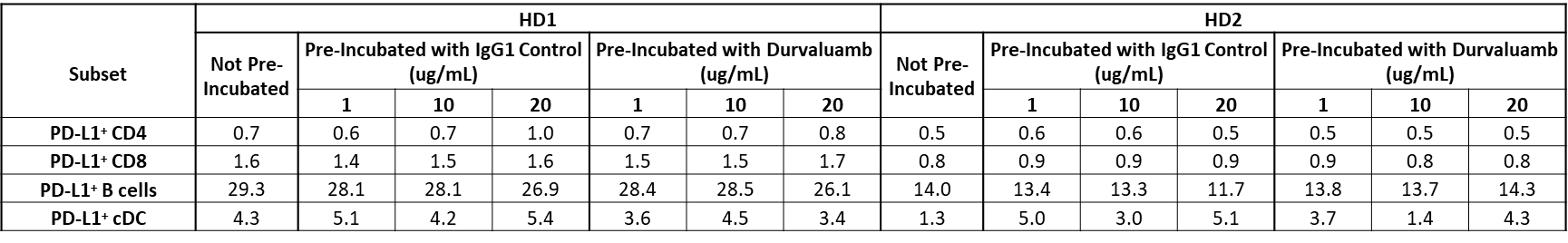
**

PD-L1 clone MIH-1 used to detect surface expression of PD-L1 in immune cell subsets does not compete for binding with durvalumab. These results demonstrate that the PD-L1 clone (MIH-1) does not compete for binding with durvalumab in PBMC and can thus be used to measure PD-L1 expression in patients treated with durvalumab.
