# Supplementary material for: Activity of durvalumab plus olaparib in metastatic castration-resistant prostate cancer in men with and without DNA damage repair mutations
Source: J Immunother Cancer. 2018 Dec 4;6:141. doi: 10.1186/s40425-018-0463-2 (PMC6280368; doi:10.1186/s40425-018-0463-2)
Supplement: Supplementary file 2 — A. Immunophenotyping of myeloid-derived suppressor cells (MDSCs). B. Statistical analysis of pharmacodynamic endpoints. (DOCX 18 kb) [file 40425_2018_463_MOESM2_ESM.docx]

**Additional file 2**

**Immunophenotyping**

The following immunophenotypic markers were used to define MDSCs: (CD3, CD19, CD56) lineage-negative HLA-DR^–^CD11b^+^CD33^+^. MDSCs were evaluated for CD40 expression. DC subsets were defined as lineage-negative HLA-DR^+^CD11c^+^CD1c^+^: CD1c^+^ mDC, lineage-negative HLA-DR^+^CD11c^+^CD141^+^: CD141 mDC, lineage-negative HLA-DR^+^CD11c^+^CD303^+^: plasmacytoid DC (pDC), DC subsets were evaluated for CD83 expression. For CD8^+^ T cells, CD4^+^ T cells, and regulatory T cells (Tregs), the following immunophenotypic markers were used: CD4^−^CD8^+^, CD8^+^ T cells; CD8^−^CD4^+^, CD4^+^ T cells; CD8^−^CD4^+^CD25^hi^Foxp3^+^, Tregs. CD8^+^ T cells and CD4^+^ T cells were evaluated for levels of expression of PD-1, CD38, TIM3, HLA-DR and Ki67. The following monoclonal antibodies were used (all from BioLegend, San Diego, CA, USA): for MDSC analysis, CD3 clone OKT3, CD56 clone MEM-188, CD19 clone HIB19, HLA-DR clone L243, CD11b clone ICRF44, CD33 clone WM53, and CD40 clone 5C3. For DC analysis, CD11c clone Bu15, CD1c clone L161, CD141 clone M80, CD303 clone 201A and CD83 clone HB15e. For T-cell subset analysis, CD8 clone SK1, CD4 clone RPA-T4, CD25 clone BC96, Foxp3 clone 206D, PD-1 clone EH12, CD38 clone HIT2, Tim-3 clone F38-2E2 and Ki67 clone B56.

Cells were incubated with Fc receptor blocking agent (Miltenyi Biotec, Bergisch Gladbach, Germany) and stained with monoclonal antibodies for 20 min at 4°C. For intracellular staining for Foxp3 and Ki67 expression, cells were fixed and permeabilized using a Fix/Perm buffer (eBiosciences, San Diego, CA, USA) according to the manufacturer’s instructions, then stained with anti-Foxp3 or anti-Ki67 antibody. Live cells were discriminated by means of LIVE/DEAD Fixable Aqua Dead Cell Stain (Life Technologies, New York, NY, USA), and dead cells were excluded from the analysis. All analyses were performed using multiparametric flow cytometry (MACSQuant; Miltenyi Biotec) and data were analyzed using FlowJo software ​v.10.0.7 (FlowJo, LLC, Ashland, OR, USA).

**Statistical analysis**

All pharmacodynamic endpoints are exploratory biomarkers. They are intended to be hypothesis-generating and require confirmation in larger prospective clinical trials. The Kaplan-Meier method was used to obtain estimates of PFS, and curves based on biomarker data divided into 2 groups were compared with a 2-tailed log-rank test. A Wilcoxon signed rank test was used to compare differences in biomarker values between pairs of time points. All *p* values are 2-tailed, not adjusted for multiple comparisons, and obtained using GraphPad Prism software v.7.0 (GraphPad Software Inc., La Jolla, CA, USA).
